# Racial Discrimination and Resting-State Functional Connectivity of Salience Network Nodes in Trauma-Exposed Black Adults in the United States

**DOI:** 10.1001/jamanetworkopen.2021.44759

**Published:** 2022-01-24

**Authors:** E. Kate Webb, Claire M. Bird, Terri A. deRoon-Cassini, Carissa N. Weis, Ashley A. Huggins, Jacklynn M. Fitzgerald, Tara Miskovich, Kenneth Bennett, Jessica Krukowski, Lucas Torres, Christine L. Larson

**Affiliations:** 1 Department of Psychology, University of Wisconsin–Milwaukee; 2 Department of Psychology, Marquette University, Milwaukee, Wisconsin; 3 Division of Trauma & Acute Care Surgery, Department of Surgery, Medical College of Wisconsin, Milwaukee; 4 Institute for Health and Equity, Department of Epidemiology, Medical College of Wisconsin, Milwaukee; 5 Brain Imaging and Analysis Center, Duke University, Durham, North Carolina; 6 VA Northern California Healthcare System, Martinez; 7 A Montana Healthcare System, Helena

## Abstract

**Importance:**

For Black US residents, experiences of racial discrimination are still pervasive and frequent. Recent empirical work has amplified the lived experiences and narratives of Black people and further documented the detrimental effects of racial discrimination on both mental and physical health; however, there is still a need for further research to uncover the mechanisms connecting experiences of racial discrimination with adverse health outcomes.

**Objective:**

To examine neurobiological mechanisms that may offer novel insight into the association of racial discrimination with adverse health outcomes.

**Design, Setting, and Participants:**

This cross-sectional study included 102 Black adults who had recently experienced a traumatic injury. In the acute aftermath of the trauma, participants underwent a resting-state functional magnetic resonance imaging scan. Individuals were recruited from the emergency department at a Midwestern level 1 trauma center in the United States between March 2016 and July 2020. Data were analyzed from February to May 2021.

**Exposures:**

Self-reported lifetime exposure to racial discrimination, lifetime trauma exposure, annual household income, and current posttraumatic stress disorder (PTSD) symptoms were evaluated.

**Main Outcomes and Measures:**

Seed-to-voxel analyses were conducted to examine the association of racial discrimination with connectivity of salience network nodes (ie, amygdala and anterior insula).

**Results:**

A total of 102 individuals were included, with a mean (SD) age of 33 (10) years and 58 (57%) women. After adjusting for acute PTSD symptoms, annual household income, and lifetime trauma exposure, greater connectivity between the amygdala and thalamus was associated with greater exposure to discrimination (*t*_(97)_ = 6.05; false discovery rate (FDR)–corrected *P* = .03). Similarly, racial discrimination was associated with greater connectivity between the insula and precuneus (*t*_(97)_ = 4.32; FDR-corrected *P* = .02).

**Conclusions and Relevance:**

These results add to the mounting literature that racial discrimination is associated with neural correlates of vigilance and hyperarousal. The study findings extend this theory by showing that this association is apparent even when accounting for socioeconomic position, lifetime trauma, and symptoms of psychological distress related to an acute trauma.

## Key Points

**Question:**

Are experiences of racial discrimination associated with altered resting-state connectivity patterns of salience network nodes?

**Findings:**

In this cross-sectional study of 102 Black adults, more experiences of racial discrimination were associated with altered connectivity of the amygdala and anterior insula, even after adjusting for annual household income, lifetime trauma exposure, and current posttraumatic stress disorder symptoms.

**Meaning:**

These findings suggest that experiencing racial discrimination is associated with modifications to known neural correlates of vigilance, suggesting a viable mechanism by which racism negatively affects mental health.

## Teaser

This cross-sectional study examines the association of racial discrimination and the connectivity of salience network nodes to offer insight into the association between racism and adverse health outcomes.

## Introduction

Racism has been deemed a public health crisis in the United States.^1^ Racial discrimination, defined as prejudice, unfair treatment, and/or violence against a marginalized racial or ethnic group, is a form of racism that can traverse different contexts. Exposure to discrimination may occur at interpersonal, environmental, and structural levels.^2,3,4^ In the United States, Black people report the highest levels of exposure to racial discrimination compared with any other racial or ethnic group.^5^ The association between racial discrimination and negative health outcomes, such as depression, anxiety, posttraumatic stress disorder (PTSD), hypertension, and heart disease, among others, has been well-established.^4,6,7,8,9,10,11,12,13,14,15,16,17,18^ Experiences of racial discrimination have also been linked to dysfunction of biological stress response systems, including greater allostatic load^19,20,21^ and shortened telomere length,^22,23,24,25^ suggesting an association with premature aging and declining health. Evidence of the association between racial discrimination and negative mental health outcomes has mostly relied on demonstrating this connection through self-reported data.^6,14,17,26,27,28,29^ However, the underlying neurobiological consequences of chronic exposure to racial discrimination has been less frequently characterized. The purpose of this study was to examine whether cumulative experiences of racial discrimination—which represent real and frequent threats—are associated with altered functional connectivity of brain regions that monitor and process threatening information.

Although the neurobiology of threat-related processes has received considerable attention, these scientific advancements (with few exceptions) have largely ignored the stressful experiences specific to racially and ethnically marginalized groups.^30,31,32,33^ There remain many questions regarding the neural consequences of chronic racial discrimination and the biological pathways between these experiences and health outcomes. One plausible pathway is that ongoing exposure to racially charged situations presents imminent social, emotional, and/or physical threats.^34,35^ The detection, monitoring, and processing of these threats naturally requires vigilance,^36,37,38^ and accordingly, threat-related brain regions are reasonably activated during and in ongoing anticipation of experiences of racism-related vigilance.^37,39^ A number of these threat-related regions are categorized as nodes in the salience network (SN; eg, amygdala and insula), which is recruited during vigilance and heighted arousal; however, the association between the SN and racial discrimination has only recently been considered.

Two studies^31,33^ have suggested that altered resting-state connectivity of nodes in the SN, such as the amygdala, insula, and connected sensory-processing regions (eg, thalamus, visual cortex), are associated with racial discrimination. Han and colleagues^33^ found differential insula connectivity in older Black adults reporting greater racial discrimination. In the context of the SN, the insula is involved in perceptions of self-awareness as well as determining the valence of external stimuli, particularly stimuli tied to social situations.^33,40,41^ Altered functional connectivity between the insula and regions implicated in vigilance (eg, intracalcarine cortex, supplementary motor area) was associated with a greater number of experiences of racial discrimination, whereas reduced connectivity between the insula and dorsolateral prefrontal cortex was associated with fewer discriminatory experiences.^33^ These vigilance regions have been implicated in other social constructs frequently associated with the experiences of racial discrimination, including behavioral response-selection to emotional stimuli and mistrust.^33^ In a racially, ethnically, and sexually diverse sample of adults (10% White, 72% Black, 23% Hispanic, 32% gay or bisexual), greater social discrimination was associated with greater spontaneous resting-state connectivity between the amygdala, another key node of the SN, and various other regions, including the insula.^31^ Interestingly, Clark and colleagues^31^ found the most robust association with social discrimination was greater connectivity between the amygdala and thalamus. In this study, and in others on stressful and traumatic experiences, aberrant resting-state functional connectivity between the amygdala and other regions in the SN (eg, anterior insula, medial prefrontal cortex, dorsal anterior cingulate cortex) has been documented.^31,42^

The SN, including the amygdala and anterior insula, has also been implicated in the symptoms observed in PTSD symptomology, specifically that of hyperarousal.^43,44,45,46,47,48^ Hyperarousal is generally characterized by feelings of persistent alertness and feeling on guard.^49^ Notably, experiences of racial discrimination are uniquely associated with symptoms of hyperarousal and vigilance,^4,37,38^ and racial discrimination predicts symptoms of PTSD.^6,14,50^ The shared experience of hyperarousal and vigilance suggests common activation of the SN^31,33,51^ and offers theoretical justification that the brain regions affected by traumatic experiences are similarly activated by racism-related vigilance. Indeed, although previous work has found novel associations between resting-state functional connectivity of SN nodes and discrimination, related symptoms, including posttraumatic stress, were not previously considered in the prediction model.^31,33^

The current study sought to replicate and advance the existing knowledge on the consequences of racism-related vigilance. Specifically, we examined the association between racial discrimination and connectivity of SN nodes (ie, the amygdala and anterior insula) during resting-state functional magnetic resonance imaging (fMRI), in the acute aftermath of traumatic injury. Given that in multiple studies,^52,53,54^ Black US residents reported the highest levels of discrimination and therefore are at high risk of racism-related poor health outcomes, we tested this question in a sample of Black adults. We enrolled Black participants after they experienced an acute traumatic injury, which allowed us to better examine whether SN vigilance systems remain associated with experiencing racial discrimination even after controlling for symptoms resulting from an acute traumatic event.

## Methods

### Participants

The current cross-sectional study was part of a larger longitudinal project examining acute posttrauma factors associated with PTSD. Individuals were recruited from the emergency department at a Midwestern level 1 trauma center in the United States between March 2016 and July 2020. Overall, 969 injured individuals treated in the emergency department were approached by the study recruitment team. Eligibility criteria were met if the individual spoke English, was aged between 18 and 65 years, could schedule a study visit within 2 weeks of the index trauma, and had experienced a traumatic injury. Participants were considered ineligible if they had a moderate to severe traumatic brain injury, suffered a spinal cord injury, or had a history of psychotic or manic symptoms. Individuals who sustained traumatic injuries resulting from suicide attempts or self-harm were also excluded. Finally, participants were required to be eligible for MRI (eg, could not be pregnant or have ferromagnetic material in the body). The described procedures were approved by the Medical College of Wisconsin institutional review board. Approximately 2 weeks post injury, participants returned for their initial study visit. At this time, individuals provided written informed consent to participate in the study. As part of a larger battery, participants completed various self-report measures and underwent neuroimaging. All individuals were financially compensated for their time. This study followed the Strengthening the Reporting of Observational Studies in Epidemiology (STROBE) reporting guideline.

Of the 215 people who were enrolled in the larger project, 198 completed the resting-state fMRI scan. One hundred and twelve participants self-reported their racial identity as Black and/or African American and had useable resting-state scans. Of those participants, 102 completed the questionnaires on lifetime exposure to racial discrimination, lifetime trauma exposure, and PTSD symptoms (administered during the same study visit). Sample demographic details can be found in Table 1.

**Table 1.** Sample Characteristics and Descriptive Statistics

Characteristics	Participants, No. (%) (N = 102)
Age, mean (SD), y	33.0 (10.4)
Gender	
Female	58 (57)
Male	44 (43)
Annual household income, $	
≤10 000	25 (25)
>10 000-20 000	19 (19)
>20 000-30 000	15 (15)
>30 000-40 000	10 (10)
>40 000-50 000	10 (10)
>50 000-60 000	6 (6)
>60 000-70 000	6 (6)
>70 000-80 000	6 (6)
>80 000-90 000	<5%
>90 000-100 000	<5%
>100 000	<5%
Mechanism of injury	
Motor vehicle crash	71 (70)
Assault/altercation	15 (14)
Other	16 (15)
Lifetime trauma	
PCL-5 score, mean (SD)	26.18 (18.0)
PEDQ item score, mean (SD)	1.93 (0.82)

Abbreviations: PCL-5, Posttraumatic Stress Disorder Checklist for *Diagnostic and Statistical Manual of Mental Disorders* (Fifth Edition); PEDQ, Perceived Ethnic Discrimination Questionnaire.

### Measures

#### Racial Discrimination

Lifetime exposure to racial discrimination was evaluated using the validated Perceived Ethnic Discrimination Questionnaire (PEDQ).^55^ This measure consists of 17 items (current sample Cronbach α = 0.93) spanning multiple settings and levels of which racial discrimination can occur. Participants were asked to quantify how frequently each item of discrimination had happened to them. Each exposure type was rated from 1 (never) to 5 (very often) and a total score was created by averaging scores from all items. Previously, greater scores were shown to be associated with baseline PTSD symptoms and predictive of chronic PTSD symptoms in this sample.^6^

#### Life Events Checklist for *DSM-5*

Lifetime trauma exposure was evaluated using the Life Events Checklist for the *Diagnostic and Statistical Manual of Mental Disorders* (Fifth Edition) (LEC-5).^56^ Sixteen different traumatic exposures are evaluated, and participants rate their experience with each event (eg, happened to them, witnessed the event, learned about the event, does not apply). The LEC ranges from 0 to 102, with higher scores indicating more exposure and closer proximity to traumatic events. A newly developed scoring method^57^ was used in which the total score was weighted according to proximity to the trauma exposure.

#### Income

Annual household income was assessed using a semicontinuous scale. A 1 indicated an annual household income of $0 to $10 000. Each 1-unit increase corresponded with a $10 000 increase in income. An 11 designated that income was greater than $100 000.

#### PTSD Checklist for *DSM-5*

Acute posttraumatic stress symptoms (ie, current PTSD symptoms related to the index trauma) were measured using the PTSD Checklist for *DSM-5* (PCL-5).^58^ In this validated self-report questionnaire, 20 items (current sample Cronbach α = 0.94) were presented which correspond to the *DSM-5* PTSD symptoms.^49^ Participants rated how much each of the symptoms (ie, items) bothered them on a scale of 1 (not at all) to 5 (extremely) since the time of the injury. A total symptom severity score was created by summing all of the items (sample range 0-73, with higher scores indicating more severe symptoms).

### MRI Data Acquisition

All neuroimaging was conducted using a Discovery MR750 3.0 Tesla scanner with a 32-channel head-coil (General Electric). High-resolution T1-weighted images were collected for coregistration using the following parameters: field of view (FOV), 240 mm; matrix, 256 × 224; slice thickness, 1 mm; 150 slices; repetition time (TR)/echo time (TE), 8.2/3.2 seconds, flip angle, 12^o^; voxel size, 1 × 0.938 × 0.938 mm. Participants underwent an 8-minute eyes-open resting-state scan, during which 240 volumes were acquired using the following parameters: FOV, 22.4 mm; matrix, 64 × 64; slice thickness, 3.5 mm; 41 sagittal slices; TR/TE, 2000/25 milliseconds; flip angle, 77°; voxel size, 3.5 × 3.5 × 3.5 mm.

### fMRI Preprocessing

Structural and resting-state images were preprocessed in the CONN toolbox version 20,^59^ with SPM version 12 and MatLab version 2019b (Mathworks). The first 3 TRs were discarded, and then images were motion-corrected using a 6-parameter linear transformation, normalized to Montreal Neurological Institute template (MNI 152) and then spatially smoothed using a 4-mm full-width-at-half-maximum kernel. During the first-level analyses, head motion parameters (and their first-order derivates) as well as white matter signal and cerebrospinal fluid signal were regressed out. If more than 20% of the resting-state volumes were scrubbed or the scan quality was deemed poor after visual inspection, the participant was removed from analyses (n = 4).

### Statistical Analysis

**Figure 1.**
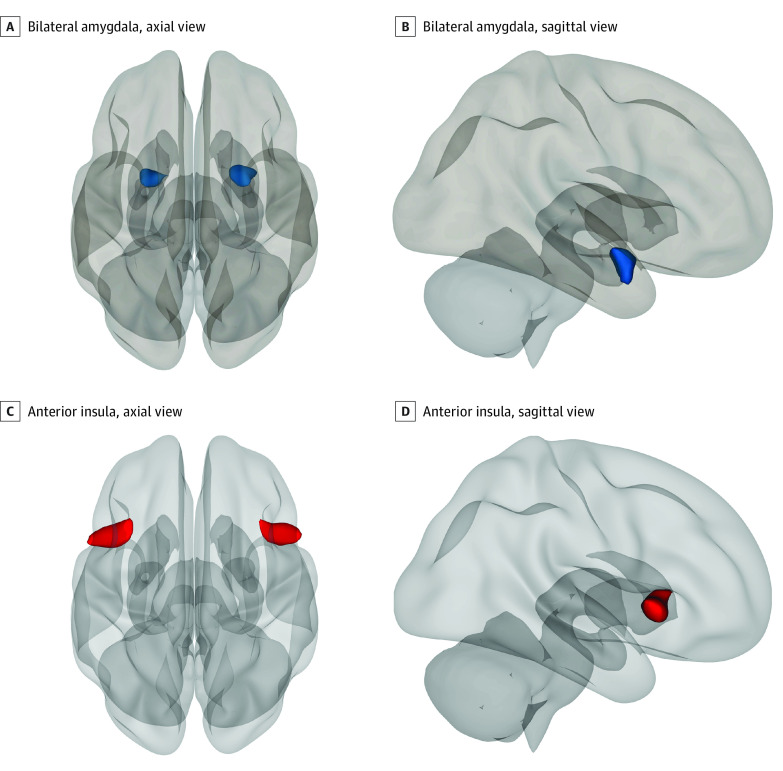
Regions Probed in the Seed-to-Voxel Connectivity Analyses

Bilateral seed regions of interests were defined using atlases in the CONN toolbox.^59^ In the toolbox, the anterior insula was functionally defined, whereas the amygdala seed was anatomically defined.^59^ We conducted 2 separate seed-to-voxel analyses (performed in CONN) in which the mean blood oxygenation level dependent signals from the bilateral amygdala (Figure 1A and B) and anterior insula (Figure 1C and D) were separately correlated with all other voxels in the brain. In group-level regression analyses, PCL-5 scores, annual household income, and lifetime trauma exposure were included as covariates. The main association of interest was that of the PEDQ mean item score on connectivity patterns.

Connectivity statistics were considered significant at a 2-tailed *P* < .05, with a height threshold of *P* < .001 uncorrected and a cluster-size threshold of an adjusted *P* < .05 false discovery rate (FDR)-corrected. The CONN toolbox was used for these analyses. Data were analyzed from February to May 2021.

## Results

Of the 102 participants (mean [SD] age, 33 [10] years; 58 [57%] women), 71 (70%) experienced a motor vehicle crash (Table 1). Pearson correlations between study measures revealed higher PCL-5 scores were significantly associated with greater exposure to racial discrimination (*r*_(100)_ = 0.49; *P* < .001) and lifetime trauma (*r*_(100)_ = 0.48; *P* < .001). PTSD symptoms were not associated with age (*r*_(100)_ = −0.06; *P* = .53) or income (*r*_(100)_ = 0.01; *P* = .90). Higher LEC-5 weighted total scores were correlated with more experiences of racial discrimination (*r*_(100)_ = 0.40; *P* < .001) but not age (*r*_(100)_ < −0.01; *P* *=* .97) or income (*r*_(100)_ = 0.09; *P* *=* .38).

### Associations of Racial Discrimination With Resting-State Connectivity Patterns

**Figure 2.**
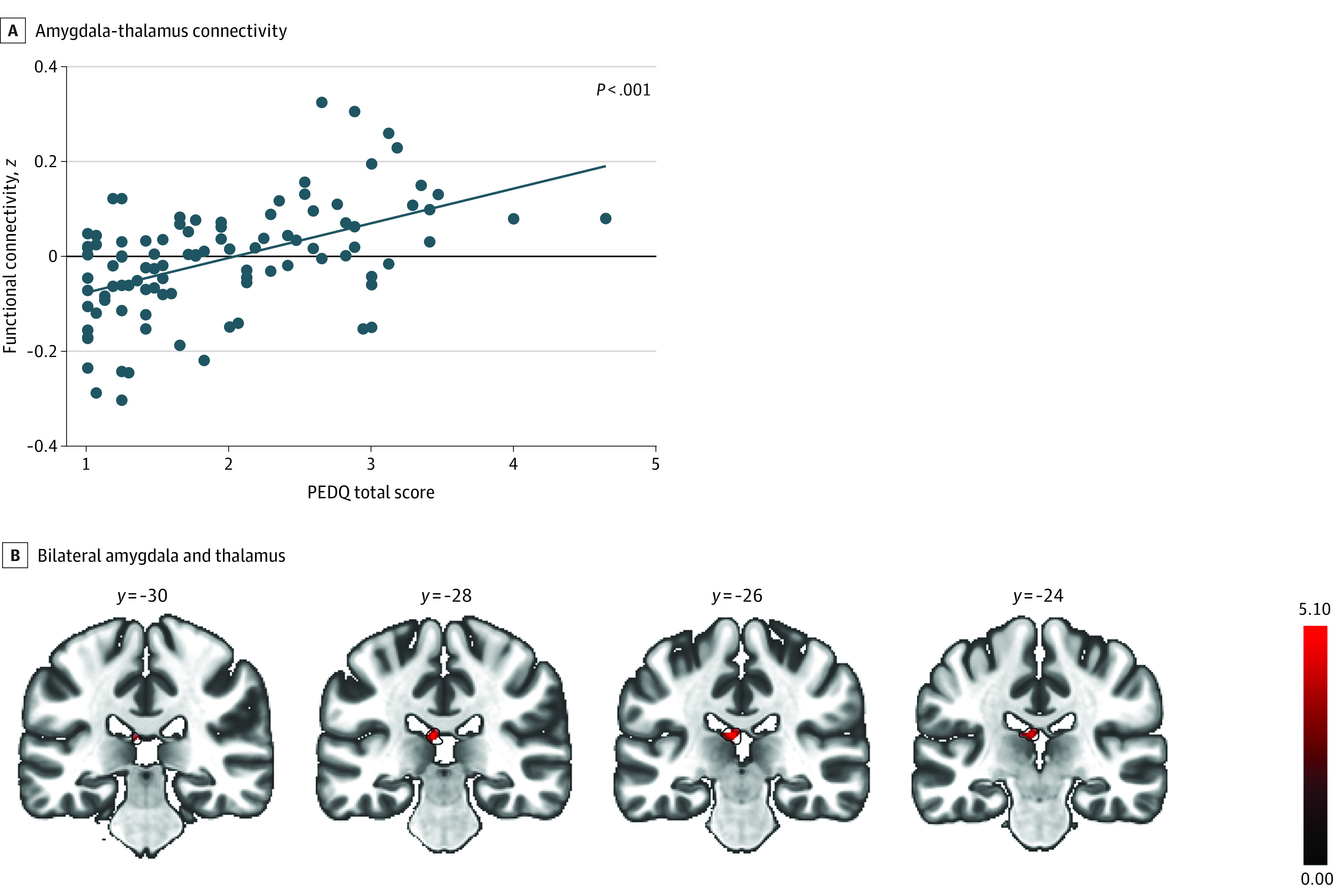
Association of Exposure to Racial Discrimination With Connectivity Between Bilateral Amygdala and Thalamus Each dot represents data from 1 participant, and the blue line is the regression line. PEDQ indicates Perceived Ethnic Discrimination Questionnaire.

**Figure 3.**
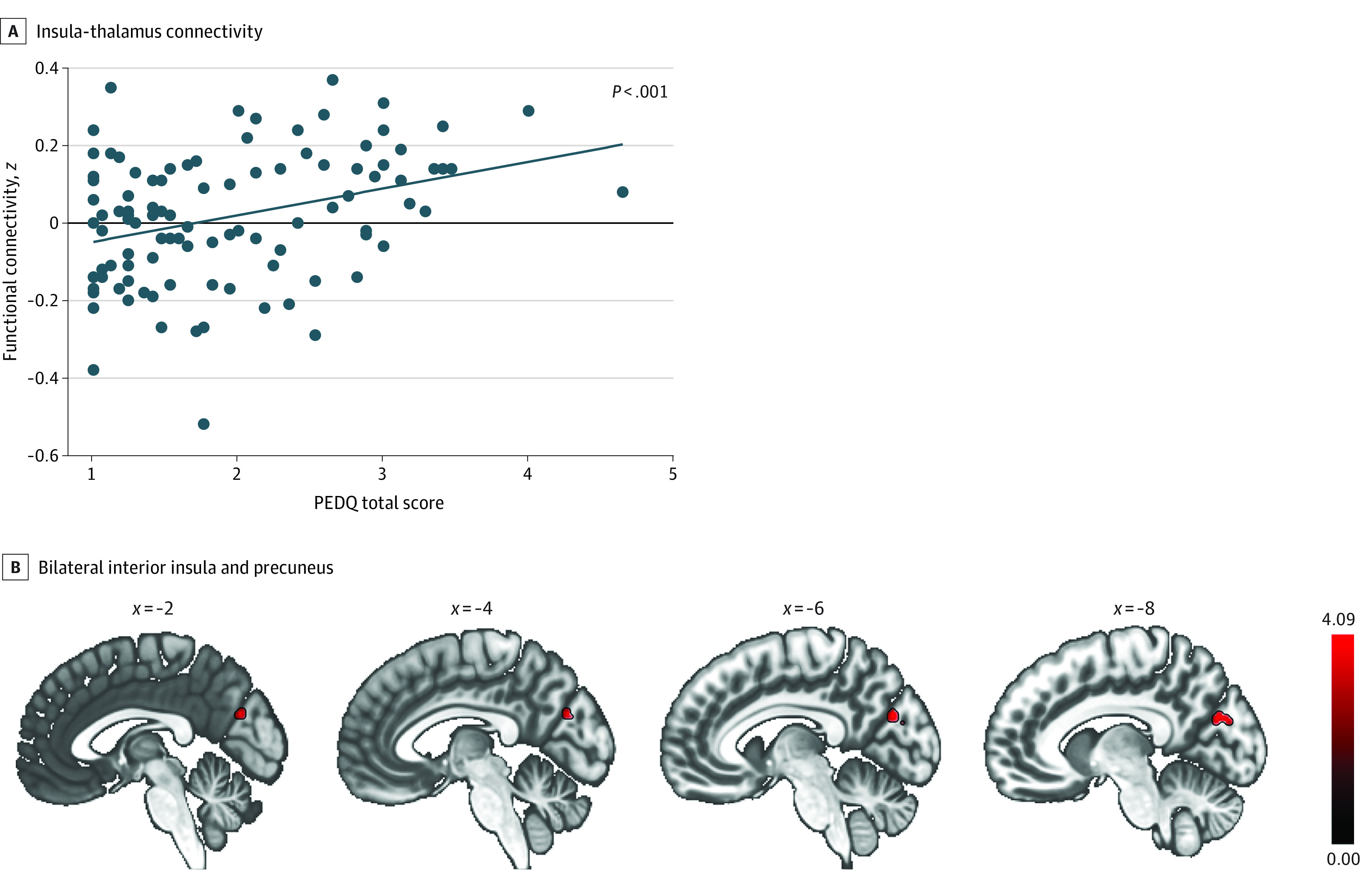
Association of Exposure to Racial Discrimination With Increased Connectivity Between Bilateral Anterior Insula and Precuneus Each dot represents data from 1 participant, and the blue line is the regression line. PEDQ indicates Perceived Ethnic Discrimination Questionnaire.

Greater exposure to racial discrimination was significantly associated with increased bilateral amygdala connectivity with the thalamus, even after adjusting for PTSD symptoms, lifetime trauma, and income (MNI coordinates x = −6; y = −26; z = 16; cluster size: *k* = 80; *t*_(97)_ = 6.05; FDR-corrected *P* = .03; Cohen *d* *=* 0.61) (Figure 2). After controlling for PCL-5 scores, income, and LEC-5 scores, greater connectivity between bilateral anterior insula and precuneus was associated with more exposure to racial discrimination (MNI coordinates: x = −6; y = −74; z= 26; cluster size: *k* = 99; *t*_(97)_ = 4.32; FDR-corrected *P* = .02; Cohen *d* *=* 0.44) (Figure 3 and Table 2).

**Table 2.** Association of Racial Discrimination With Greater Connectivity of Regions Implicated in Emotion Processing

ROI	Contrast	Brain region	Voxels, No.	FDR-corrected *P* value	Peak MNI coordinates		
					X	Y	Z
Amygdala	Positive	Thalamus	95	.02^a^	−6	−26	16
Anterior insula	Positive	Cuneus	78	.03^a^	−8	−74	24

Abbreviations: FDR, false-discovery rate; MNI, Montreal Neurological Institute; ROI, region of interest (seed region).

^a^ FDR-corrected *P* < .05 (N = 102). Models were adjusted for posttraumatic stress disorder symptoms, lifetime trauma (Life Events Checklist weighted scores), and income.

## Discussion

We evaluated the association between experiences of racial discrimination and resting-state functional connectivity of the SN (ie, amygdala and anterior insula) in Black US residents recently exposed to a trauma. By analyzing this association in a sample of individuals who recently experienced a traumatic injury, we attempted to evaluate the neural consequences of experiencing discrimination while adjusting for the contributions related to an acute trauma. These findings replicated recent work, demonstrating experiences of discrimination are associated with increased connectivity between threat-activated brain regions, particularly those responsible for initial processing of salient stimuli.^31,32,33^

Greater amygdala connectivity with the thalamus, a crucial intermediary between sensory systems and affective/cognitive regions, has been previously correlated with more exposure to social discrimination.^31^ Similar patterns of connectivity are demonstrated during anticipation of uncertain threats,^60^ a category which discriminatory experiences may frequently fall in. We position the current findings within studies on threat appraisal, such as that by Jenks et al,^61^ which suggest greater amygdala and thalamus connectivity reflects a greater sensitivity to threatening situations. Importantly, increased amygdala-thalamus connectivity at rest may be reflective of an adaptative coping strategy to mentally prepare for threats associated with racial discrimination, which are horrifically persistent for Black US residents living in a White-centric society.^62^ Attempting to use and maintain coping strategies to combat racism may be associated with increased demands on psychosocial resources (ie, cognitive and affective processes^11^), thereby leading to neural vulnerabilities or an increased biological sensitivity exploited by acute trauma.^39^ Supporting this theory are findings from Fani et al^32^: in Black women with trauma exposure, more experiences of racial discrimination were significantly associated with greater activation of visual regions and the ventromedial prefrontal cortex (a node in the SN) during trauma-relevant images compared with neutral images.

Increased SN connections with sensory regions is also frequently characterized as evidence for vigilance, integration of external information, and internal arousal.^43,46,63,64,65^ Given the precuneus’s role in mental imagery, greater insula-precuneus connectivity at rest aligns with the theory that discrimination is associated with increased preparedness for biologically relevant information and general arousal.^66^ The precuneus, a node of the default mode network, is also involved in self-elicited recollection of past experiences.^67,68^ In fact, mental imagery of negative or fear stimuli elicits activation of both the insula and precuneus.^69^ The anterior insula and precuneus also underly aspects of self-awareness,^40,70,71^ which could relate to the heighted awareness a Black person may feel when navigating through the White-centric United States. A strong sense of self, belonging, and commitment to a racial or ethnic community seems to have a protective function against the psychological consequences of discrimination.^62,72,73,74^ In addition, Mekawi and colleagues^75^ found that more active race-based coping strategies (eg, talking about experiences of racism and doing something to address it) rather than more passive (eg, accept the experience and do not talk about it) also helped to buffer the association between discrimination and mental health outcomes.

Alterations to SN nodes have been associated health outcomes, including symptoms of PTSD,^43,64,76^ general anxiety disorder,^63,77,78^ and even peripheral inflammation.^79^ Although we did not directly examine the association between discrimination and future health outcomes, within the context of this broader literature, the current work supports a discrimination-perpetual vigilance-altered SN–health outcome pathway. Indeed, in the literature on PTSD, aberrations in amygdala-thalamus activation and connectivity are broadly thought to correlate with emotion dysregulation.^80,81^ Difficulty regulating emotions or using adaptive coping strategies may stem from challenges in appropriately identifying and early processing of emotionally relevant stimuli.^82^ Perhaps this is one mechanism by which racism instigates symptoms of anxiety and depression, although more work is needed in this area.

These findings are situated in and constrained by the complexities of racial discrimination, trauma exposure, and PTSD symptoms. Greater previous exposure to traumatic and stressful experiences increases the risk of PTSD development after an acute trauma.^57^ In tandem, racial discrimination increases risk of PTSD.^15,83,84^ Previously, in this sample, we found that racial discrimination was associated with baseline posttraumatic stress symptoms and nonremitting (6 months post injury) PTSD symptoms.^6^ Thus, acute trauma exposure and racial discrimination together present a compounded risk factor that may affect neural connectivity and lead to subsequent PTSD.^84^ In fact, racial discrimination in the absence of an acute trauma is associated with PTSD symptoms.^85^ Although we included PTSD symptom severity scores as a covariate in the models, adjusting for these scores alone may not fully capture the neural consequences of experiencing a traumatic injury. Acute posttraumatic stress symptoms are also correlated with racial discrimination,^6,14^ which presents the possibility that the PTSD symptoms assessed, although the measures explicitly queried the index trauma, may also capture experiences of discrimination. We did not specifically probe racism-related vigilance or hyperarousal; future directions include administering surveys specifically designed to measure the construct,^37^ rather than relying on surveys developed for PTSD assessment.

In the current study, we evaluated racial discrimination in the absence of any protective factors that may buffer the harmful consequences of racism. Unfortunately, we did not capture any racism-related coping strategies^39^ that may help prevent negative mental health symptoms from developing. This is certainly a limitation; identifying resilience factors is critical because they represent ideal targets that therapeutic interventions can help bolster. For example, Forsyth and Carter^39^ chronicled how different racial identity attitudes and racism-related coping strategies were associated with varying degrees of psychological symptoms. In the ongoing discussion and future work on racism and mental health outcomes, it is imperative we move forward by evaluating protective factors which may mitigate the neural consequences of racism.

Conceptualizing experiences of racial discrimination as a both a form of psychological trauma and as a shared risk factor for psychopathology represents an important shift in the White-centric understanding of the consequences of racism.^84^ The threat associated with racial discrimination increases racism-related vigilance.^37,38^ In this study, we observed an association between racial discrimination and the amygdala and anterior insula, 2 SN regions underlying threat-related vigilance. This is a viable mechanism by which these experiences can contribute to PTSD and also possibly alter neural functioning even independent of an acute trauma (as shown in other studies^31,32,33^). Moreover, racism-related vigilance may confound work on vigilance tied to an acute trauma, therefore, we (and other researchers^14,86^) recommend assessing racial discrimination in studies on trauma and stress.

### Limitations

This study has additional limitations that temper its generalizability. It used a homogeneous sample, such that majority of the participants experienced a motor vehicle crash. This may result in a unique profile of posttrauma symptomatology and/or brain connectivity. However, given that traumatically injured, Black US residents are generally understudied, the findings from the current study remain particularly valuable and informative. Previous work in the same sample^6^ demonstrated that discrimination was associated with acute posttraumatic stress and predictive of future PTSD. This further supports theories characterizing racial discrimination as a form of racial trauma.^4,5,7,84^ Based on this conceptualization, it is challenging to fully disentangle the neural impact of past racial trauma, current PTSD symptoms, and lifetime nonracialized trauma. Nevertheless, we attempted to statistically control for these associations by including LEC and PCL-5 scores in our models. Notably, we conducted cross-sectional analyses and therefore cannot draw conclusions about causal relationships. Future work should explore these relationships longitudinally and test whether altered SN connectivity mediates the association between racial discrimination and nonremitting PTSD. Examining the association of racial discrimination, in the context of trauma, with both brain function and structure is necessary to elucidate how discrimination affects the neurobiological underpinnings of PTSD and drives posttrauma health disparities.

## Conclusions

Consistent with the limited existing work^31,32,33^ on the associations between racial discrimination and the brain, in this study we found these experiences were associated with alterations in functional connectivity in individuals exposed to trauma. Taken together, this accumulating evidence emphasizes the gravity of racism as a public health crisis. While neuroscience research can be used to underscore the biological consequences of racism and help test factors that may buffer against these effects, the overarching goals should be to provide culturally informed treatment to Black Americans with PTSD symptoms and combat racism—interpersonal and structural—in US society.
